# The importance of stem cell engineering in head and neck oncology

**DOI:** 10.1007/s10529-016-2163-7

**Published:** 2016-06-24

**Authors:** Wojciech Barczak, Pawel Golusiński, Lukasz Luczewski, Wiktoria M. Suchorska, Michal M. Masternak, Wojciech Golusiński

**Affiliations:** 1 Department of Head and Neck Surgery, The Greater Poland Cancer Centre, Poznań University of Medical Sciences, Garbary 15 Str., 61-866 Poznan, Poland; 2 Radiobiology Lab, Department of Medical Physics, The Greater Poland Cancer Centre, Garbary 15 Str., 61-866 Poznan, Poland; 3 Burnett School of Biomedical Sciences, College of Medicine, University of Central Florida, 6900 Lake Nona Blvd., Orlando, FL 32827 USA; 4 Department of Electroradiology, Poznan University of Medical Sciences, Garbary 15 Str., 61-866 Poznan, Poland

**Keywords:** Head and neck cancer, HESC, Induced pluripotent stem cells, Mesenchymal stem cells, Stem cell tissue engineering

## Abstract

Head and neck squamous cell carcinoma is the sixth leading cause of cancer worldwide. The most common risk factors are carcinogens (tobacco, alcohol), and infection of the human papilloma virus. Surgery is still considered as the treatment of choice in case of head and neck cancer, followed by a reconstructive surgery to enhance the quality of life in the patients. However, the widespread use of artificial implants does not provide appropriate physiological activities and often cannot act as a long-term solution for the patients. Here we review the applicability of multiple stem cell types for tissue engineering of cartilage, trachea, vocal folds and nerves for head and neck injuries. The ability of the cells to self-renew and maintain their pluripotency state makes them an attractive tool in tissue engineering.

## Introduction

Head and neck cell carcinoma (HNSCC), the sixth leading cause of cancer worldwide, represents approx. 4 % of all malignant tumors and 5 % mortality (Ferlay et al. 2008). Among head and neck cancers, over 90 % are squamous cell carcinomas, ascending from the epithelial cells that line the mucosal surfaces. Among the risk factors, smoking increases the risk up to ten times more as compared with non-smokers, and alcohol represents an independent risk factor (Hashibe et al. 2007). The combined effect of smoking tobacco and alcohol consumption causes multiplicative risk (Hashibe et al. 2009). Other molecular determinants of HNSCC are mutations and polymorphisms in epidermal growth factor receptor, PI3 K (phosphatidylinositol 3-kinase), AKT (protein kinase B, PKB), and p16 (cyclin-dependent kinase inhibitor 2A) pathways. These aberrations cause anomalous survival signaling. There is also altered regulation of genes directing apoptosis, cell cycle, epithelial-mesenchymal transition (EMT), hypoxia response (SLC2A1), metabolism and insulin-like growth factor (IGF) pathway signal in HNSCC (Zhi et al. 2014, 2015).

Malignant tumors of the head and neck area are different in terms of clinical outcome and prognosis and depend on the histological diagnosis and anatomical site. To maximize the radicalization of therapy, a combination of treatments involving the local procedures (surgery, radiotherapy) and chemotherapy are commonly used. Such proceedings provide the best chance to cure the patients and increase their overall survival rate (Dietz et al. 2012). A radical treatment approach requires combined therapy surgery (50 %) commonly followed by post-operative radiotherapy (45 %) and chemotherapy (5 %) or radiochemotherapy. The qualification of patients for the treatment depends on evidence based on the medical status of the patients, histopathology, anatomical site of the tumor and the Tumor-Node-Metastasis classification.

Surgery is still the treatment of choice in majority of the cases of head and neck cancer and a follow-up reconstructive surgery becomes imperative.

The reconstruction surgery of head and neck region is complicated and challenging due to the requirements of multicomponent reconstructions and adjuvant therapy. Tissue engineering has been rapidly developed as an emerging research field with relevant implications for the improvement of the course of treatment.

In the clinical conditions, wherein a disease leads to tissue damage, an organ and tissue transplantation provides an alternative way to restore and improve the quality of life of the patients. Unfortunately, tissue and organ transplantation is severely limited by donor shortage and possible immune rejection. The currently used artificial scaffolds including artificial joints cannot replicate all the functions of the human tissues, therefore limiting their long-term viability. Furthermore, artificial tissues may not necessarily be suitable for integration into the host tissues (Chapekar 2000).

One of the cell source used in tissue engineering are stem cells: human embryonic stem cells (hES) and induced pluripotent stem cells (Murry and Keller 2008). Their unique characteristic of self-renewal and maintenance of the pluripotency state can be used in the therapy of many disorders.

One of the procedures to obtain embryonic cells (ES) is the nuclear transfer of the somatic cell into an oocyte with previously removed nuclear genetic material. The mechanism of parthenogenesis, i.e. transformation of the oocyte in the embryo without fertilization using a defined chemical protocol, is also used (Condic 2008). In 2006, Kazutoshi Takahashi and Yamanaka (2006) indicated the possibility of receiving stem cells from murine fibroblasts by introducing four transcription factors: Oct3/4 (octamer-binding transcription factor 3/4), Sox2 [SRY (sex determining region Y)-box 2], Klf4 (Kruppel-like factor 4), and c-Myc (Avian myelocytomatosis virus oncogene cellular homolog). The reprogrammed cells had similar morphology and genes expression profile as that of the embryonic cells. Those cells were named as induced Pluripotent Stem Cells (iPSC) (Takahashi and Yamanaka 2006). iPSC were also obtained from human fibroblasts (Park et al. 2008; Takahashi et al. 2007). The use of iPSC cells allows marginalization of two key problems associated with the ES cells: transplant rejections and ethical controversies concerning use of the embryos. Thus, the iPSC cells can be used for transplantation in patients with damaged spinal nerve, or in the treatment of neurodegenerative diseases (e.g. Parkinson’s disease). This technology is also considered to be breakthrough for the modeling of inherited genetic diseases (Huntington’s disease, Alzheimer’s disease) (Carpenter et al. 2009; Nishikawa et al. 2008).

With advancement in technologies, there is a growing potential for using iPSC and ES cells in tissue engineering as a component of constructs engineered at the cellular and basic tissue levels (cartilage, bones, esophagus, trachea, vessels, nerve) (Hwang et al. 2008; Jungebluth et al. 2011; Leydon et al. 2013).

Despite the effectiveness of stem cell-based therapies, a number of disadvantages could be listed. One of those is genetic instability during in vitro culture (chromosomal rearrangements, aneuploidy, defective DNA methylation), which results in decreased differentiation capacity and increased proliferation rate. Moreover, changes in culture conditions in vitro could contribute to epigenetic instability, and long-term culture may increase the risk of these anomalies. Another concern is the process of reprogramming, which may lead to the creation of genetically unstable induced pluripotent stem cells. High costs of reprogramming and increase risk of teratoma formation are also an important issues (Suchorska et al. 2016).

The aim of this paper is to review the usefulness of stem cells in the tissue engineering of head and neck oncology.

## Biomaterials in tissue engineering

The materials used in tissue engineering must possess certain characteristics such as lack of toxicity (especially the lack of oncogenic potential), biocompatibility, and sterility. The mechanical requirements such as plasticity, stability or flexibility are also important. The aim of tissue engineering is to restore, defend (avoid disease progression) and/or improve the function of the damaged tissues and organs. The material must allow for cell adhesion, supplying the growth factors, and excretion of the metabolic products (Yang et al. 2001; Walgenbach et al. 2001). The biological tissue substitutes for engineering must contain three critical components: cells capable of populating tissue substitute and generating appropriate constituents of the novel tissue, biomimetic scaffolds, and environmental signals (i.e. growth factors, small molecules, or physical factors facilitating direct cells differentiation toward the appropriate lineage) (Butler et al. 2000; Brunger et al. 2014). Biomimetic scaffolds allow for the infiltration of cells and are able to support extracellular matrix (ECM) accumulation and organization. These scaffolds can mimic the architecture of the native extracellular matrix at the nanoscale level (nanofibers and nanopores), which provides the initial space for regeneration of new tissue (Wei and Ma 2008). In traditional tissue engineering methods, cells seeded into a scaffold with biocompatible and biodegradable properties (especially scaffolds capable of supporting three-dimensional tissue formation) are used. The result of this approach is the populating of cells in the scaffold and creation of their own extracellular matrix (Lu et al. 2013; Correia et al. 2013; Macchiarini 2006).

## Cartilage

Cartilage has a very limited regenerative capacity of its own due to the lack of blood supply and progenitor cells migrating to the tissue, which makes it a perfect candidate for tissue engineering. The current treatments are based on transplantation of autologous chondrocytes and osteochondral allografts (Bartlett et al. 2005; Sheyn et al. 2010; Augustyniak et al. 2015). In this regard, pluripotent stem cells have arisen as an attractive cell source for repairing large cartilage defects after the primary surgery of the laryngeal cancer. The great potential of stem cells for self-renewal and chondrogenic differentiation also offer great potential in the regeneration of necrotic cartilage after radiation (Keeney et al. 2011). A cartilage-like matrix can form in vivo without teratoma formation, which is crucial for clinical application. Hwang et al. (2008) characterized a human embryonic-derived mesenchymal progenitor population without cell sorting, hESCd-MSCs, and demonstrated their capacity of expansion and multilineage differentiation. They also demonstrated that the chondrocyte-secreted morphogenetic factors may be a promising method for inducing in vivo commitment and cartilaginous tissue formation. Thus, the described methods may be useful for the successful treatment of articular cartilage defects, avoiding growth factors addition or gene transduction (Hwang et al. 2008). Transplanted hESC-derived chondrogenic cells can maintain a long-term viability with no evidence of tumorigenicity, thereby providing a safe, highly-efficient and practical strategy of applying hESCs for cartilage tissue engineering (Toh et al. 2010; Lach et al. 2014).

Attention is now being focused on adult mesenchymal stem cells (MSCs) from the bone marrow. MSCs play a pivotal role as support cells for haematopoiesis. They function as a reservoir of cells to regenerate various types of mesenchymal tissues (Gerson 1999). The number of MSCs in the bone marrow comes to 10^6^, and decreases with age. Surface antigens (i.e. CD44, CD71, CD90, CD106, CD120a, and CD124) are highly expressed in the MSCs cells. However no expression of haematopoietic lineage markers such as CD14, CD34, and CD45 was confirmed (Campagnoli et al. 2001). The potential of MSCs to generate cartilage has been evaluated (Risbud and Sittinger 2002). The most established marrow-based technique is called microfracture. The damaged area is perforated below the subchondral plate, allowing the blood to flow and clot in the microfractures. The blood clot contains a relatively high proportion of marrow-derived MSCs with high chondrogenic differentiation potential, which subsequently produce a scar tissue more related with the fibrocartilage than the actual cartilage (Mitchell and Shepard 1976; Kreuz et al. 2006). Improvements in the technique use growth factors and anti-inflammatory agents, and also biodegradable scaffold materials that improve the cartilage repair volume, composition, and stability as planned by the authors in the ongoing clinical trials (Berthiaume et al. 2011).

Csaki et al. (2008) summarized the different selected methodologies for cartilage repair and the potential usefulness of MSCs. The MSCs could differentiate along the adipogenic, osteogenic, and chondrogenic lineages as a dermis isolated adult stem cells (DIAS cells). Passaging these cells in chondrogenic medium and forming them into self-assembled tissue-engineered constructs caused upregulation of collagen type II and COMP gene expression. Incorporating transforming growth factor β1 (TGF-β1) or bone morphogenetic protein 2 (BMP-2) to DIAS constructs led to a higher sulfated glycosaminoglycan content. Additionally, the TGF-β1 treatment caused a significant increase in the compressive properties and construct contraction. Contrary to these results, BMP-2 treatment resulted in the largest constructs with decreasing compressive properties. The obtained results showed that the DIAS can be used for cartilage tissue engineering strategies, and as a cell source for other mesenchymal tissues (Sanchez-Adams and Athanasiou 2012).

Tissue engineering combined with gene therapy results in a modified and novel tissue engineering technology. Guo et al. (2007) achieved repair of the articular cartilage defects after the implantation of genetically engineered cartilage using TGF-β1-transfected MSCs and chitosan. The TGF-β1-gene was transferred into MSCs using liposomes. The chondrogenic differentiation of MSCs in vitro, and the expressions of chondrogenic ECM and metalloproteinases were also investigated (Guo et al. 2007).

## Trachea

Besides cartilage, other tissues are also an attractive target for tissue engineering. In the head and neck area, primary cancers of the trachea are rare but are characterized by a high mortality rate. The gold standard of treatment for these tumors is surgical resection with primary reconstruction (Macchiarini 2006). However, an inoperable size at the time of diagnosis excludes the chances of surgical resection. Macchiarini et al. (2008) reported the first fully tissue engineered tracheal transplantation with a non-immunogenic human donor trachea derived from the bone marrow mesenchymal stem and respiratory cells. This approach was limited by the lack of an appropriate size of the donor’s organs (Macchiarini et al. 2008). Jungebluth et al. (2011) reported the clinical transplantation of the tracheobronchial airway with a stem-cells-seeded bioartificial nanocomposite, which had clear anastomoses, lined with a vascularised neomucosa, and was partly covered by a nearly healthy epithelium. Post-operatively, the authors detected a recruitment of the peripheral cells presenting increased mesenchymal stromal cell phenotype, and upregulation of epoetin receptors, antiapoptotic genes, and miR-34 and miR-449 biomarkers.

Tracheal engineering replacements are used as a hopeful and simple strategy in tissue engineering in vivo. This method is based on the intraoperatively replacement of the native airway using a scaffold as a biological incubator and specific environmental signals. In situ tissue regeneration (in patient’s body as a natural bioreactor for cell proliferation and migration) guarantees increasing host stem cells deployment inside the implanted biopolymers (Baiguera et al. 2010). Conconi et al. (2009) demonstrated that the use of bioengineered matrix provides native biological and biomechanical features. The research was based on results that reported acellular matrices maintain the expression of angiogenic proteins (bFGF and TGF-β) and applied robust angiogenic responses. Expression of proteins induced by human bioengineered trachea was shown both for the in vitro and in vivo angiogenic responses. In this study, the bioartificial system consisting of VEGF-loaded porous silica gel and myoblasts cultured on acellular diaphragmatic matrix was implanted to repair a surgically created diaphragmatic defect in Lewis rats (Conconi et al. 2009). The system based on scaffold bioreactor developed for growing tissue engineered trachea and the effect of the fluid flow on producing trachea-like neotissue were investigated by Lin et al. (2009). To obtain an increased cell proliferation, the GAG and collagen content in the constructs and rotation culture was used. In the future, this stage could represent improvement in applying tissue engineering techniques to regenerate tracheas (Lin et al. 2009).

Adipose-derived stem cells are another source of stem cells for trachea tissue engineering. Using adult derived stem cells eliminates the ethical issue of hES. Kobayashi et al. (2010) used artificial grafts made from collagen sponge with a spiral polypropylene stent and mesh for patients with tracheal resection. The authors investigated the potential of gingival fibroblasts and adipose derived stem cells (ASCs) as autologous transplanted cells in combination with artificial graft for tracheal epithelial regeneration. Here, the authors form the bioengineered scaffold, which contain adipose derived stem cells with a thick induced epithelium (Kobayashi et al. 2010). Gingival fibroblasts assisted tracheal epithelial cell growth, cell differentiation, and epithelial reconstruction, resulting in pseudostratified ciliated epithelium that was similar to the normal tracheal epithelium (Kobayashi et al. 2007). Thus, ASCs could be used as autologous transplant cells for tracheal regeneration (Suzuki et al. 2008). The application of ASCs has several advantages, including simplicity of isolation, relative abundance, rapid expansion, and multipotency (Zuk et al. 2002).

## Vocal folds

Progress in reconstructive tissue engineering has contributed to the development of methods for repair of vocal folds. Damage to the vocal cords is a major problem after partial laryngectomy. Kanemaru et al. (2005) investigated the destiny of implanted autologous bone marrow derived stromal cells (BSCs) containing mesenchymal stem cells. The inserted BSCs were traced in order to determine the type of tissues resulting from the injected site of the vocal fold. The implanted cells were alive in the host tissues and presented positive expression of markers for the epithelial tissue (keratin) and muscle (desmin), which demonstrate that MSC differentiate into various cell types (Kanemaru et al. 2005).

Different groups analyzed the short-term viscoelastic and histologic properties of scarred rabbit vocal folds after the injection of human mesenchymal stem cells, as well as the percentage of MSC survival. The treated scarred vocal folds showed persistent MSC. Injected MSC into canine injured vocal folds reported better wound healing and improved viscoelastic parameters as compared to the control group treated by collagen injection only (Hertegård et al. 2006). The hES derived epithelial cells with the primary vocal fold fibroblasts under defined conditions can stimulate the production of progenitor cells into vocal fold epithelial-like cells and create a basement membrane. Leydon et al. (2013) showed that obtained hESC derived epithelial cells demonstrated expression of vocal fold epithelial markers: keratin 13 and 14, as well as gap junctions and desmosomes. This proves that the hESC derived epithelial progenitor cells can be engineered to create an in vitro model of vocal fold mucosa (Leydon et al. 2013).

## Nerves

The neuronal cells are obtained by differentiation of the bone marrow MSC in engineered nanofibrous scaffolds. This provides potential for bionanomaterial cell transplantation therapy of neurodegenerative diseases and injuries of the nervous system (recurrent laryngeal nerve, facial nerve). Prabhakaran et al. (2009) investigated the potential of those cells for neuronal differentiation in vitro on poly(l-lactic acid)-co-poly-(3-caprolactone)/collagen (PLCL/Coll) nanofibrous scaffolds. The authors suggested the potential application of mesenchymal stem cells towards nerve regeneration. Both MSC and iPSC have the potential for treatment of traumatic injury of the nervous system. Kuo and Chen (2013) performed a differentiation of iPSCs into neuron-lineage cells in inverted colloidal crystal (ICC) scaffolds containing alginate, poly(γ-glutamic acid), and surface peptide (CSRARKQAASIKVAVSADR). There was reduced expression of stage-specific embryonic surface antigen-1 and the raised expression of β III tubulin in differentiated iPSCs by culturing ICC constructs with CSRARKQAASIKVAVSADR. Based on this research, the increase of iPSCs differentiation toward neurons for nerve tissue engineering by the induction with CSRARKQAASIKVAVSADR in ICC topography was established (Kuo and Chen 2013). The previously mentioned mesenchymal stromal cells, adipose derived stem cells and bone marrow mesenchymal stem cells, as multipotent adult stem cells derived from adipose and bone marrow tissue, has an unlimited prospective in nerve tissue engineering. Data from Xu et al. (2008) demonstrated that Schwann cell (SC)-like cells, showing a spindle-like shape and expressing glial markers GFAP and S100, and differentiated from the adipose-derived stem cells are able to form myelin. This suggests their ability for the repair of nerve damages. The same conclusions were demonstrated by Kingham et al. (2007) who indicated a regenerative potential of adipose tissue derived Schwann cells in the peripheral nerve injuries treatment.

Neural stem cells (NSCs) hold great promise for cell therapies and tissue engineering. NSCs occurs in the nervous system and can differentiate into neurons, astrocytes, and glial cells. They were used in tissue-engineered studies due to their multipotent capacity, high immigration ability, and low immunogenicity. Caprini et al. (2013) synthesized two LDLK-12-based self-assembling peptides functionalized with KLPGWSG peptide (KLP and Ac-KLP). The peptides supported differentiation of murine neural stem cells in vitro. The authors also demonstrated that the Ac-KLP SAP-based (acetylated at the *N*-terminus LDLK-12 core with the KLPGWSG peptide self-assembling peptides) scaffold enhances the neuronal differentiation of neural stem cells. These indications make KLPGWSG and the Ac-KLP peptide promising candidates to be used in stem cell therapies for nervous regeneration (Caprini et al. 2013). Cheng et al. (2013) demonstrated a functional peptide-based scaffold. The extended IKVAV sequence enhanced signal transduction to direct neural stem cells adhesion leading towards neuronal differentiation. RADA (16)-IKVAV self-assembling peptide hydrogel improved the survival rate of condensed NSCs and reduced the formation of glial astrocytes (Cheng et al. 2013). Ni et al. (2014) revealed that the microporous/micropatterned poly(d,l-lactic acid) nerve conduits containing bioactive acidic fibroblast growth factor 1 could be fabricated by micropatterning techniques, open plasma activation, and immobilization, which, combined with aligned stem cells, may synergistically contribute to the regeneration of the severely damaged peripheral nerve.

## Conclusions

The hESCs, iPSCs and other types of stem cells have great potential in tissue engineering. These cells can differentiate between a variety of cell types like cartilage, neural, or muscle cells. This unlimited source, and introduced improvements of the use of stem cells with a proper scaffold component, increases the chances of for greater usage in the future. Promising results indicate that stem cells have therapeutic potential for tissue engineering applications. However, further studiessss are required to estimate the safety of the therapeutic use of stem cells in humans.

## References

[CR1] Augustyniak E, Trzeciak T, Richter M, Kaczmarczyk J, Suchorska W (2015). The role of growth factors in stem cell-directed chondrogenesis: a real hope for damaged cartilage regeneration. Int Orthop.

[CR2] Baiguera S, Jungebluth P, Burns A, Mavilia C, Haag J, De Coppi P (2010). Tissue engineered human tracheas for in vivo implantation. Biomaterials.

[CR3] Bartlett W, Skinner JA, Gooding CR, Carrington RW, Flanagan AM, Briggs TW (2005). Autologous chondrocyte implantation versus matrix-induced autologous chondrocyte implantation for osteochondral defects of the knee: a prospective, randomised study. J Bone Jt Surg Br.

[CR4] Berthiaume F, Maguire TJ, Yarmush ML (2011). Tissue engineering and regenerative medicine: history, progress, and challenges. Annu Rev Chem Biomol Eng.

[CR5] Brunger JM, Huynh NP, Guenther CM, Perez-Pinera P, Moutos FT, Sanchez-Adams J (2014). Scaffold-mediated lentiviral transduction for functional tissue engineering of cartilage. Proc Natl Acad Sci USA.

[CR6] Butler DL, Goldstein SA, Guilak F (2000). Functional tissue engineering: the role of biomechanics. J Biomech Eng.

[CR7] Campagnoli C, Roberts IA, Kumar S, Bennett PR, Bellantuono I, Fisk NM (2001). Identification of mesenchymal stem/progenitor cells in human first-trimester fetal blood, liver, and bone marrow. Blood.

[CR8] Caprini A, Silva D, Zanoni I, Cunha C, Volontè C, Vescovi A (2013). A novel bioactive peptide: assessing its activity over murine neural stem cells and its potential for neural tissue engineering. Nat Biotechnol.

[CR9] Carpenter MK, Frey-Vasconcells J, Rao MS (2009). Developing safe therapies from human pluripotent stem cells. Nat Biotechnol.

[CR10] Chapekar MS (2000). Tissue engineering: challenges and opportunities. J Biomed Mater Res.

[CR11] Cheng TY, Chen MH, Chang WH, Huang MY, Wang TW (2013). Neural stem cells encapsulated in a functionalized self-assembling peptide hydrogel for brain tissue engineering. Biomaterials.

[CR12] Conconi MT, Bellini S, Teoli D, de Coppi P, Ribatti D, Nico B (2009). In vitro and in vivo evaluation of acellular diaphragmatic matrices seeded with muscle precursors cells and coated with VEGF silica gels to repair muscle defect of the diaphragm. J Biomed Mater Res.

[CR13] Condic ML (2008). Alternative sources of pluripotent stem cells: altered nuclear transfer. Cell Prolif.

[CR14] Correia SI, Pereira H, Silva-Correia J, Van Dijk CN, Espregueira-Mendes J, Oliveira JM (2013). Current concepts: tissue engineering and regenerative medicine applications in the ankle joint. J R Soc Interface.

[CR15] Csaki C, Schneider PR, Shakibaei M (2008). Mesenchymal stem cells as a potential pool for cartilage tissue engineering. Ann Anat.

[CR16] de Souza Lucena EE, Guzen FP, de Paiva Cavalcanti JRL, Barboza CAG, do Nascimento Júnior ES, Cavalcante Jde S (2014). Experimental considerations concerning the use of stem cells and tissue engineering for facial nerve regeneration: a systematic review. J Oral Maxillofac Surg.

[CR17] Dietz A, Boehm A, Wichmann G, Niederwieser D, Dietzsch S, Fuchs M (2012). Multimodal laryngeal preservation: current data-based opinion. HNO.

[CR18] Ferlay J, Shin HR, Bray F, Forman D, Mathers C, Parkin DM (2008). Estimates of worldwide burden of cancer in 2008: GLOBOCAN. Int J Cancer.

[CR19] Gerson SL (1999). Mesenchymal stem cells: no longer second class marrow citizens. Nat Med.

[CR20] Guo CA, Liu XG, Huo JZ, Jiang C, Wen XJ, Chen ZR (2007). Novel gene-modified-tissue engineering of cartilage using stable transforming growth factor-beta1-transfected mesenchymal stem cells grown on chitosan scaffolds. J Biosci Bioeng.

[CR21] Hashibe M, Brennan P, Benhamou S, Castellsague X, Chen C, Curado MP (2007). Alcohol drinking in never users of tobacco, cigarette smoking in never drinkers, and the risk of head and neck cancer: pooled analysis in the International Head and Neck Cancer Epidemiology Consortium. J Natl Cancer Inst.

[CR22] Hashibe M, Brennan P, Chuang SC, Boccia S, Castellsague X, Chen C (2009). Interaction between tobacco and alcohol use and the risk of head and neck cancer: pooled analysis in the International Head and Neck Cancer Epidemiology Consortium. Cancer Epidemiol Biomark Prev.

[CR23] Hertegård S, Cedervall J, Svensson B, Forsberg K, Maurer FH, Vidovska D (2006). Viscoelastic and histologic properties in scarred rabbit vocal folds after mesenchymal stem cell injection. Laryngoscope.

[CR24] Hwang NS, Varghese S, Lee HJ, Zhang Z, Ye Z, Bae J (2008). In vivo commitment and functional tissue regeneration using human embryonic stem cell-derived mesenchymal cells. Proc Natl Acad Sci USA.

[CR25] Jungebluth P, Alici E, Baiguera S, Le Blanc K, Blomberg P, Bozóky B (2011). Tracheobronchial transplantation with a stem-cell-seeded bioartificial nanocomposite: a proof-of-concept study. Lancet.

[CR26] Kanemaru S, Nakamura T, Yamashita M, Magrufov A, Kita T, Tamaki H (2005). Destiny of autologous bone marrow-derived stromal cells implanted in the vocal fold. Ann Otol Rhinol Laryngol.

[CR27] Keeney M, Lai JH, Yang F (2011). Recent progress in cartilage tissue engineering. Curr Opin Biotechnol.

[CR28] Kingham PJ, Kalbermatten DF, Mahay D, Armstrong SJ, Wiberg M, Terenghi G (2007). Adipose-derived stem cells differentiate into a Schwann cell phenotype and promote neurite outgrowth in vitro. Exp Neurol.

[CR29] Kobayashi K, Suzuki T, Nomoto Y, Tada Y, Miyake M, Hazama A (2007). Potential of heterotopic fibroblasts as autologous transplanted cells for tracheal epithelial regeneration. Tissue Eng.

[CR30] Kobayashi K, Suzuki T, Nomoto Y, Tada Y, Miyake M, Hazama A (2010). A tissue-engineered trachea derived from a framed collagen scaffold, gingival fibroblasts and adipose-derived stem cells. Biomaterials.

[CR31] Kreuz PC, Steinwachs MR, Erggelet C, Krause SJ, Konrad G, Uhl M (2006). Results after microfracture of full-thickness chondral defects in different compartments in the knee. Osteoarthr Cartil.

[CR32] Kuo YC, Chen CW (2013). Inverted colloidal crystal scaffolds with induced pluripotent stem cells for nerve tissue engineering. Colloids Surf B.

[CR33] Lach M, Trzeciak T, Richter M, Pawlicz J, Suchorska W (2014). Directed differentiation of induced pluripotent stem cells into chondrogenic lineages for articular cartilage treatment. J Tissue Eng.

[CR34] Leydon C, Selekman JA, Palecek S, Thibeault SL (2013). Human embryonic stem cell-derived epithelial cells in a novel in vitro model of vocal mucosa. Tissue Eng Part A.

[CR35] Lin CH, Hsu SH, Huang CE, Cheng WT, Su JMA (2009). Scaffold-bioreactor system for a tissue-engineered trachea. Biomaterials.

[CR36] Lu T, Li Y, Chen T (2013). Techniques for fabrication and construction of three-dimensional scaffolds for tissue engineering. Int J Nanomed.

[CR37] Macchiarini P (2006). Primary tracheal tumours. Lancet Oncol.

[CR38] Macchiarini P, Jungebluth P, Go T, Asnaghi MA, Rees LE, Cogan TA (2008). Clinical transplantation of a tissue-engineered airway. Lancet.

[CR39] Mitchell N, Shepard N (1976). The resurfacing of adult rabbit articular cartilage by multiple perforations through the subchondral bone. J Bone Jt Surg Am.

[CR40] Murry CE, Keller G (2008). Differentiation of embryonic stem cells to clinically relevant populations: lessons from embryonic development. Cell.

[CR41] Nishikawa S, Goldstein RA, Nierras CR (2008). The promise of human induced pluripotent stem cells for research and therapy. Nat Rev Mol Cell Biol.

[CR42] Park IH, Zhao R, West JA, Yabuuchi A, Huo H, Ince TA (2008). Reprogramming of human somatic cells to pluripotency with defined factors. Nature.

[CR43] Prabhakaran MP, Venugopal JR, Ramakrishna S (2009). Mesenchymal stem cell differentiation to neuronal cells on electrospun nanofibrous substrates for nerve tissue engineering. Biomaterials.

[CR44] Risbud MV, Sittinger M (2002). Tissue engineering: advances in in vitro cartilage generation. Trends Biotechnol.

[CR45] Sanchez-Adams J, Athanasiou KA (2012). Dermis isolated adult stem cells for cartilage tissue engineering. Biomaterials.

[CR46] Sheyn D, Mizrahi O, Benjamin S, Gazit Z, Pelled G, Gazit D (2010). Genetically modified cells in regenerative medicine and tissue engineering. Adv Drug Deliv Rev.

[CR47] Suchorska WM, Augustyniak E, Łukjanow M (2016). Genetic stability of pluripotent stem cells during anti-cancer therapies. Exp Ther Med.

[CR48] Suzuki T, Kobayashi K, Tada Y, Suzuki Y, Wada I, Nakamura T (2008). Regeneration of the trachea using a bioengineered scaffold with adipose-derived stem cells. Ann Otol Rhinol Laryngol.

[CR49] Takahashi K, Yamanaka S (2006). Induction of pluripotent stem cells from mouse embryonic and adult fibroblast cultures by defined factors. Cell.

[CR50] Takahashi K, Tanabe K, Ohnuki M, Narita M, Ichisaka T, Tomoda K (2007). Induction of pluripotent stem cells from adult human fibroblasts by defined factors. Cell.

[CR51] Toh WS, Lee EH, Guo XM, Chan JK, Yeow CH, Cao T (2010). Cartilage repair using hyaluronan hydrogel-encapsulated human embryonic stem cell-derived chondrogenic cells. Biomaterials.

[CR52] Walgenbach KJ, Voigt M, Riabikhin AW, Andree C, Schaefer DJ, Galla TJ (2001). Tissue engineering in plastic reconstructive surgery. Anat Rec.

[CR53] Wei GB, Ma PX (2008). Nanostructured biomaterials for regeneration. Adv Funct Mater.

[CR54] Xu Y, Liu L, Li Y, Zhou C, Xiong F, Liu Z (2008). Myelin-forming ability of Schwann cell-like cells induced from rat adipose-derived stem cells in vitro. Brain Res.

[CR55] Yang S, Leong KF, Du Z, Chua CK (2001). The design of scaffolds for use in tissue engineering. Part I. Traditional factors. Tissue Eng.

[CR56] Zhi X, Lamperska K, Golusinski P, Schork NJ, Luczewski L, Golusinski W (2014). Expression levels of insulin-like growth factors 1 and 2 in head and neck squamous cell carcinoma. Growth Horm IGF Res.

[CR57] Zhi X, Lamperska K, Golusinski P, Schork NJ, Luczewski L, Kolenda T (2015). Gene expression analysis of head and neck squamous cell carcinoma survival and recurrence. Oncotarget.

[CR58] Zuk PA, Zhu M, Ashjian P, De Ugarte DA, Huang JI, Mizuno H (2002). Human adipose tissue is a source of multipotent stem cells. Mol Biol Cell.

